# Targeting TM4SF1 exhibits therapeutic potential via inhibition of cancer stem cells

**DOI:** 10.1038/s41392-022-01177-7

**Published:** 2022-10-14

**Authors:** Guang Chen, Xiaofei She, Yanxin Yin, Junxian Ma, Yaqun Gao, Hua Gao, Huanlong Qin, Jianmin Fang

**Affiliations:** 1 Cancer Center and Research Institute of Intestinal Diseases, Shanghai Tenth People’s Hospital, School of Life Sciences and Technology and School of Medicine, Tongji University, Shanghai, PR China; 2 Shanghai Key Laboratory of Signaling and Disease Research, School of Life Sciences and Technology, Tongji University, Shanghai, PR China; 3 Biomedical Research Center, Tongji University Suzhou Institute and Department of Neurology, Tongji Hospital, Tongji University, Shanghai, PR China

**Keywords:** Cancer stem cells, Cancer therapy, Metastasis

**Dear Editor**,

Cancer stem cells (CSCs) are thought to be responsible for cancer initiation, growth, recurrence, metastasis, and drug resistance.^1^ Therefore, targeting CSCs is an effective therapeutic approach for cancer.^2^ However, there are few CSC-specific targets with functional extracellular domains for the development of antibody drugs.^3^ Our published paper demonstrated that transmembrane 4 L six family member 1 (TM4SF1) coupled discoidin domain receptor tyrosine kinase 1 (DDR1) under collagen I stimulation activated JAK2-STAT3 signaling. This noncanonical DDR1 signaling sustained the manifestation of CSC traits by inducing *SOX2* and *NANOG* expression and drove multiorgan metastases.^4^

In this study, we demonstrated that TM4SF1 was a cell membrane marker of CSCs, and monoclonal antibodies (mAb) targeting functional extracellular domain of TM4SF1 inhibited CSCs. Immunohistochemistry staining of 16 types of cancers and adjacent normal tissues showed that TM4SF1 was highly expressed on the cancer cell membrane but undetectably expressed on normal cells (Fig. 1a and Supplementary Fig. 1a). Then, we examined the relationship between TM4SF1 and CSCs. There were more CD44^high^/CD24^low^ (the currently known CSC marker) cells among TM4SF1^high^ MDA-MB-231 human breast cancer cells than that among TM4SF1^low^ MDA-MB-231 cells (Fig. 1b and Supplementary Fig. 1b). TM4SF1^high^ MDA-MB-231 cells and H460 human lung cancer cells upon serial passage formed more tumor spheres than the corresponding TM4SF1^low^ cells (Fig. 1c and Supplementary Fig. 1c, d). Similar results were obtained in various human cancer cell lines, including breast cancer cell lines (lung metastatic MDA-MB-231 and MDA-MB-453), melanoma cell lines (A375 and A2058), and lung cancer cell lines (H2030 and H1975) (Supplementary Fig. 1e–j). Moreover, pluripotency factors were upregulated in TM4SF1^high^ cells of various human cancer cell lines (Supplementary Fig. 1k, l). These results suggest that TM4SF1 is a cell membrane marker of CSCs.

**Fig. 1:**
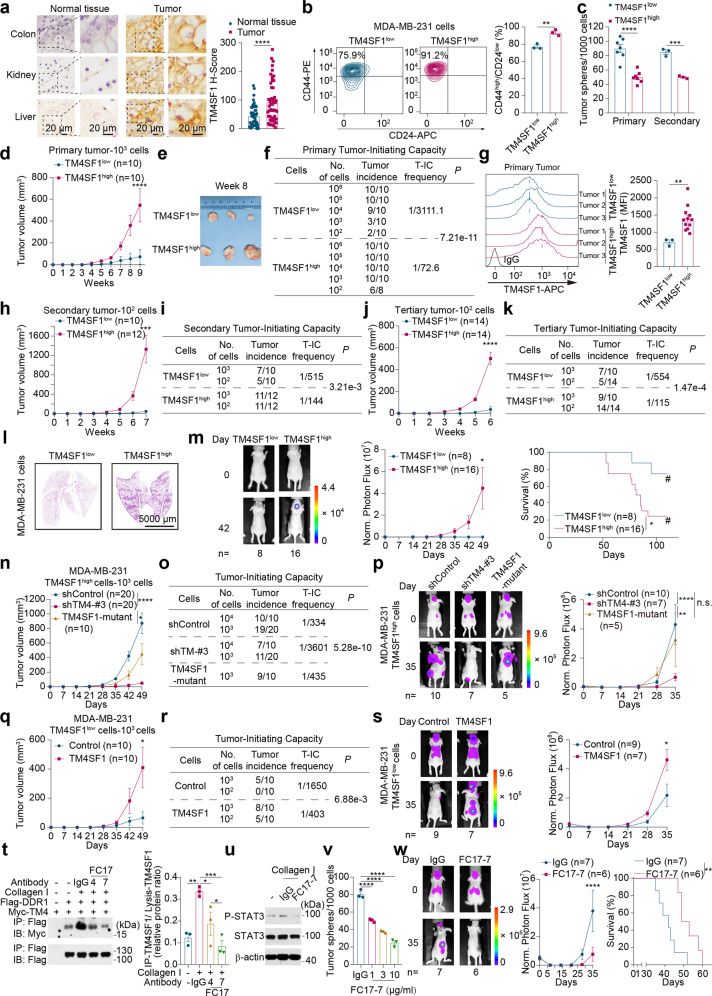
TM4SF1 is a novel molecular target for CSC therapy. **a** Representative immunohistochemical images and staining intensities of TM4SF1 expression in clinical samples of three types of organ cancer with the adjacent normal tissue (colon, kidney, and liver). Representative immunohistochemical images of other 13 types of organ cancer are shown in Fig. S1a. **b** Flow cytometric analysis of the CD44^high^/CD24^low^ expression level in TM4SF1^low^ and TM4SF1^high^ MDA-MB-231 cells (three independent experiments). **c** The sphere-forming capacity of TM4SF1^high^ cells was determined by serial tumor sphere assays (1000 cells/well). Cells were cultured with 30 μg/ml collagen I for 14 days. The primary spheres were dissociated and subjected to secondary tumor sphere assays (three independent experiments). See Supplementary Fig. S1c for representative images of spheres. **d**–**k** Serial limiting dilution transplantation assay indicated that TM4SF1 was critical for the stemness of cancer cells. Primary tumor volume at a dilution of 10^3^ cells (**d**). Representative tumor images on Day 56 at a dilution of 10^4^ cells (**e**). Primary tumor-initiating capacity (**f**). Flow cytometric analysis of TM4SF1 expression on the cell membrane of tumor cells from digested primary tumors, at least three independent experiments (**g**). Secondary tumor volume at a dilution of 10^2^ cells (**h**). Secondary tumor-initiating capacity (**i**). Tertiary tumor volume at a dilution of 10^2^ cells (**j**). Tertiary tumor-initiating capacity (**k**). (The n-values denote the number of tumors per group.) **l** Representative images of H&E staining of lung metastases from the primary transplantation assay. See Supplementary Fig. S3a for quantification (three independent experiments). **m** Bioluminescence imaging (left panels), quantification (middle panels) of metastases and overall survival (right panels) of BALB/c nude mice xenografted with TM4SF1^low^ or TM4SF1^high^ MDA-MB-231 cells by intracardiac injection (1000 cells). (The n-values denote the number of mice per group.) **n**–**p** Silencing of TM4SF1 in TM4SF1^high^ MDA-MB-231 cells inhibited the tumor-initiating and metastatic capacities. Tumor volume (The n-values denote the number of tumors per group.) (**n**). Tumor-initiating capacity (**o**). Bioluminescence imaging (left panels) and quantification (right panels) of metastases in BALB/c nude mice xenografted with shControl, shTM4-#3 or TM4SF1-mutant cells by intracardiac injection (1 × 10^5^ cells) (The n-values denote the number of mice per group.) (**p**). **q**–**s** Overexpression of TM4SF1 in TM4SF1^low^ MDA-MB-231 cells enhanced the tumor-initiating and metastatic capacities. Tumor volume (The n-values denote the number of tumors per group.) (**q**). Tumor-initiating capacity (**r**). Bioluminescence imaging (left panels) and quantification (right panels) of metastases of BALB/c nude mice xenografted with control or TM4SF1-overexpressing cells by intracardiac injection (1 × 10^5^ cells) (The n-values denote the number of mice per group.) (**s**). **t** The coimmunoprecipitation assay showed that the interaction of TM4SF1 and DDR1 was blocked by monoclonal antibodies. See Supplementary Fig. S6d for lysates (three independent experiments). **u** Western blot analysis showed that activation of STAT3 was blocked by the monoclonal antibody FC17-7. See Supplementary Fig. S6i for quantification (three independent experiments). **v** The sphere-forming capacity of cells treated with FC17-7 at the indicated concentration was determined by a tumor sphere formation assay. MDA-MB-231 cells (1000 cells/well) were cultured with IgG or FC17-7 at the indicated concentration and stimulated with collagen I (30 μg/ml) for 14 days. See Supplementary Fig. S7c for representative images of spheres (three independent experiments). **w** FC17-7 inhibited cancer metastasis. Bioluminescence imaging (left panels), quantification (middle panels) of metastases and overall survival (right panels) of BALB/c nude mice implanted with MDA-MB-231 cells by intracardiac injection (1 × 10^5^ cells). IgG or FC17-7 (10 mg/kg) was administered intraperitoneally once every 3 days (FC17-7) from day −1 to the death of mice (The n-values denote the number of mice per group.). Throughout the figure, data are presented as the mean ± s.e.m. values. *P* values were determined by an unpaired two-tailed Student’s *t* test with Welch’s correction [(**a**, **b**, **g**)] or one-way ANOVA with uncorrected Fisher’s LSD test (**t**) and (**v**) or two-way ANOVA with uncorrected Fisher’s LSD test [(**c**, **d**, **h**, **j**, **m**, **n**, **p**, **q**, **s**, **w**)] or the log-rank test [(**m**, **w**)]. *P < 0.05; **P < 0.01; ***P < 0.001; ****P < 0.0001, n.s. not significant and #, end of the experiment

Furthermore, we utilized the serial limiting dilution transplantation assay to determine the frequency of tumor-initiating cells (T-IC) (Supplementary Fig. 2a). TM4SF1^high^ MDA-MB-231 cells injected into mice exhibited more rapid tumor growth, higher T-IC frequency, and shorter latency periods than TM4SF1^low^ MDA-MB-231 cells (Fig. 1d–f and Supplementary Fig. 2b, c). Notably, TM4SF1 expression was maintained in primary tumors formed from TM4SF1^high^ or TM4SF1^low^ cells (Fig. 1g), indicating that CSCs stably maintain TM4SF1-high expression. Moreover, the expression of stemness markers, such as CD44^high^/CD24^low^, CD133, *NANOG*, *POU5F1*, and *SOX2* (Supplementary Figs. 1k, 2d, e), was higher in primary tumors formed from TM4SF1^high^ MDA-MB-231 cells than in those formed from TM4SF1^low^ MDA-MB-231 cells, indicating that TM4SF1-high expression stably maintained CSC traits. To examine whether the stemness of TM4SF1^high^ cells can be stably passaged to subsequent generations, secondary and tertiary transplantation assays were performed. TM4SF1^high^ cells exhibited more rapid tumor growth, higher T-IC frequency and shorter latency periods (Fig. 1h–k and Supplementary Fig. 2f, g, i, j) than TM4SF1^low^ cells. Moreover, fluorescence-activated cell sorting (FACS) analysis showed that TM4SF1 expression was maintained in the secondary and tertiary tumors formed from TM4SF1^high^ and TM4SF1^low^ cells, respectively (Supplementary Fig. 2h, k). Similar results were obtained in A2058 cells (Supplementary Fig. 2l–n). These results indicate that TM4SF1^high^ cells have CSC characteristics and that these characteristics were stably maintained and passaged to subsequent generations.

Since CSCs are also responsible for metastasis, we examined whether TM4SF1 expression affected metastasis. Interestingly, more metastases were developed in the lungs of mice orthotopically injected with TM4SF1^high^ MDA-MB-231 cells, and multiorgan metastases were promoted and the survival times were decreased in the mice intracardially injected with TM4SF1^high^ MDA-MB-231 cells (Fig. 1l, m and Supplementary Fig. 3a). Similar results were obtained in A2058 and H2030 cells (Supplementary Fig. 3b, c). These findings demonstrate that TM4SF1^high^ cells have higher metastatic ability than TM4SF1^low^ cells.

To examine whether high TM4SF1 expression is necessary for TM4SF1^high^ cells to possess and maintain the CSC function, we silenced TM4SF1 in TM4SF1^high^ MDA-MB-231 cells and found reductions in sphere formation, tumor growth, T-IC frequency, and metastasis (Fig. 1n–p and Supplementary Fig. 4a–d, g). Moreover, TM4SF1 depletion prolonged latency periods and survival times after orthotopic injection (Supplementary Fig. 4e, f, h, i). In contrast, this suppression was reduced by TM4SF1 overexpression in shTM4#3 cells. To further examine whether high TM4SF1 expression is a sufficient condition for the possession and maintenance of CSC characteristics, we overexpressed TM4SF1 in TM4SF1^low^ MDA-MB-231 cells and found that TM4SF1 enhanced stemness, as indicated by sphere formation, tumor growth, T-IC frequency, and metastasis, and shortened latency periods and survival times (Fig. 1q–s and Supplementary Fig. 5a–h). Similar results were obtained in A2058 cells (Supplementary Figs. 4j–m, 5i, j). Collectively, these results reveal that high TM4SF1 expression is a necessary and sufficient condition for the possession and maintenance of CSC characteristics. Therefore, TM4SF1 may be a therapeutic target for specifically eliminating CSCs.

Our previous study showed that the interaction between TM4SF1 and DDR1 on the cell membrane mediated multiorgan metastases. To further identify the interaction domain of TM4SF1, coimmunoprecipitation assays (coIP) were performed, and we found that the interaction site was the extracellular loop 1 (ECL1) of TM4SF1 (Supplementary Fig. 6a, b). Then, we used ECL1 of TM4SF1 as the antigen, screened thousands of mAbs, and obtained a series of functional mAbs (Supplementary Fig. 6c). The functional mAbs blocked the interaction of TM4SF1 and DDR1. Among these mAbs, FC17-7 exhibited the best blocking activity (Fig. 1t and Supplementary Fig. 6d), and FACS analysis showed that the binding capacity of FC17-7 was dose-dependent (Supplementary Fig. 6e). Furthermore, FC17-7 blocked collagen-induced activation of JAK2-STAT3 signaling in MDA-MB-231 cells (Fig. 1u and Supplementary Fig. 6g, i). Similar results were obtained in 293FT and A549 human lung cancer cells (Supplementary Fig. 6f, h, j). These results demonstrate that FC17-7 inhibits JAK2-STAT3 signaling activation by blocking the interaction between ECL1 of TM4SF1 and DDR1.

Next, we examined whether mAbs targeting TM4SF1 inhibited CSC function. mAbs targeting TM4SF1 abrogated the ability of MDA-MB-231 cells to form tumor spheres (Fig. 1v and Supplementary Fig. 7a, c). Similar results were obtained in H460 cells (Supplementary Fig. 7b). Interestingly, FC17-7 intensively inhibited sphere formation in TM4SF1^high^ MDA-MB-231 cells but not in TM4SF1^low^ MDA-MB-231 cells (Supplementary Fig. 7d), indicating that FC17-7 specifically inhibits CSCs but not non-CSCs. Similar results were obtained in TM4SF1^high^ A2058 and A375 cells (Supplementary Fig. 7e, f). Moreover, we found that pluripotency factors were downregulated by FC17-7 (Supplementary Fig. 7g).

We then assessed the therapeutic potential of mAbs in a metastasis model. Surprisingly, breast cancer metastasis was significantly inhibited by FC17-7, and survival time was prolonged by mAb treatment (Fig. 1w and Supplementary Fig. 7h–j). The mAbs were nontoxic to mice because there was no significant difference in weight (Supplementary Fig. 7k). These results show that the mAb FC17-7 is a potential anti-CSC drug.

TM4SF1 is a cell membrane marker of CSCs, and its functional extracellular domain also enhances CSC function. Thus, TM4SF1 is a natural and excellent target for anti-CSC drugs. By binding to ECL1 of TM4SF1, FC17-7 blocked the interaction between TM4SF1 and DDR1, suppressed JAK2-STAT3 signaling, and reduced *SOX2* and *NANOG* expression. Furthermore, FC17-7 inhibited tumor sphere formation in vitro and breast cancer metastasis in vivo, suggesting that FC17-7 is a potential anti-CSC drug for cancer therapy. We are attempting to its clinical translation without falling behind the competition with Angiex, who is developing an antibody drug conjugate against TM4SF1.^5^

## Supplementary information

Supplementary information

## Data Availability

All data are available upon reasonable request to the corresponding author.
