# Supplementary material for: Targeting TM4SF1 exhibits therapeutic potential via inhibition of cancer stem cells
Source: Signal Transduct Target Ther. 2022 Oct 14;7:350. doi: 10.1038/s41392-022-01177-7 (PMC9561108; doi:10.1038/s41392-022-01177-7)
Supplement: Supplementary file 1 — Supplementary information [file 41392_2022_1177_MOESM1_ESM.docx]

Supplementary Materials for

Targeting TM4SF1 exhibits therapeutic potential via inhibition of cancer stem cells

Guang Chen^1,2,4^, Xiaofei She^1,2,4^, Yanxin Yin^2,3,4^, Junxian Ma^1,2,4^, Yaqun Gao^1,2^, Hua Gao^1,2,*^, Huanlong Qin^1,*^, and Jianmin Fang^2,3,*^

Correspondence to: gaoh@tongji.edu.cn, qinhuanlong@tongji.edu.cn, and [jfang@tongji.edu.cn](mailto:jfang@tongji.edu.cn)

**This file includes:**

Materials and Methods

Figures. S1 to S7

**Materials and Methods**

**Cell lines**

Human breast cancer cell lines (MDA-MB-231, MDA-MB-453 and MCF7), human melanoma cell lines (A375 and A2058), and the human lung cancer cell line (A549) from the American Type Culture Collection (ATCC), and 293FT cell line from Thermo Fisher Scientific (Waltham, MA, USA) were cultured in DME-HG medium supplemented with 10% FBS (04-001-1ACS, Biological Industries, Kibbutz Belt HaEmek, Israel), 2 mM L-GLN (21051-024, Thermo Fisher Scientific), and 100 U/ml penicillin/0.1 mg/ml streptomycin (C0222, Beyotime Biotech, Shanghai, China). Human lung cancer cell lines (H460, H2030 and H1975) from ATCC were cultured in RPMI 1640 medium supplemented with 10% FBS, 2 mM L-GLN, and 100 U/ml penicillin/0.1 mg/ml streptomycin. For bioluminescent imaging, MDA-MB-231 cells were infected with TGL vector encoding GFP and firefly luciferase.^1^ H2030 and A2058 cells were infected with the TL vector encoding tdTomato and firefly luciferase.^2^ All human cell lines were authenticated by short tandem repeat (STR) analysis provided by Bio-Research Innovation Center Suzhou, SIBCB, CAS, and mycoplasma contamination was routinely detected.

**Plasmids**

TdTomato-luciferase was subcloned into the pCDH lentivirus vector and verified by sequencing. Myc-TM4SF1 and Flag-DDR1 were subcloned into the pBABE-puro, pQCXIP or pQCXIN (Clontech, San Diego, CA, USA) retrovirus vectors. The Myc-CD81-TM4SF1-ECL1 chimera, Myc-CD81-TM4SF1-ECL2 chimera, Myc-CD81-TM4SF1-ECL1&2 chimera, Myc-TM4-CD81-ECL1 chimera, Myc-TM4-CD81-ECL2 chimera, and Myc-TM4-CD81-ECL1&2 chimera were generated by PCR using complementary primers and subcloned into the pQCXIP vector. Constructs encoding shRNAs against human TM4SF1 (#2, TRCN 0000000287 and #3, TRCN 0000000288, Sigma, St. Louis, MO, USA) were generated by cloning the corresponding short hairpin sequences into the pLKO.1 vector. The shTM4SF1-#3 resistant TM4SF1 cDNA (264-282) was replaced by CGCCATGCTGAGCAGCGTG and generated by PCR using complementary primers.

**Mice**

Mice were housed under specific pathogen-free (SPF) conditions in the animal facility of Tongji University. All animal experiments were approved by the Institutional Animal Care and Use Committee of Tongji University. MDA-MB-231, A2058 and H2030 cells were xenografted into 5- to 7-week-old (15-20 g) BALB/c nude mice purchased from the SLAC Laboratory Animal Center (Shanghai, China). Mice were age and sex-matched and randomized where appropriate.

**Bioluminescent imaging**

Mice were anesthetized with isoflurane and injected retro-orbitally with 1.5 mg of D-luciferin (LUCK-1G, Gold Biotechnology, St. Louis, MO, USA) at the indicated times. Mice were imaged in a NightOWL II LB 983 (Berthold, Bad Wildbad, Germany) chamber, and Indigo^TM^ software (Berthold) was used to record and analyze the metastasis data.

**Xenograft tumor studies**

For serial limiting dilution transplantation assays, the indicated numbers of MDA-MB-231 cells and their derivatives were suspended in 30 μl DME-HG and injected into the No. 4 mammary glands of 5- to 7-week-old female BALB/c nude mice. The indicated numbers of A2058 cells and their derivatives were suspended in 30 μl DME-HG and subcutaneously inoculated into 5- to 7-week-old male nude mice. Tumor growth was monitored weekly by taking measurements of tumor length (L) and width (W). Tumor volume was calculated using the formula πLW^2^/6. For serial transplantation, xenograft tumors were digested using Trypsin (27250-018, Thermo Fisher Scientific) and used for secondary or tertiary tumor initiation assays. The frequency of T-ICs was calculated using the Extreme Limiting Dilution Analysis software (http://bioinf.wehi.edu.au/software/elda/).

**Cancer metastasis assay *in vivo***

Mice were anesthetized with isoflurane. The indicated numbers of MDA-MB-231, A2058 and H2030 cells and their derivatives were suspended in 100 μl PBS and injected into the left ventricle of mice. Bioluminescence imaging was used to verify successful injection and to monitor metastatic outgrowth. For antibody treatment, FC17-4 (10 mg/kg, every other day) or FC17-7 (10 mg/kg, every three days) was administered intravenously from day -1 to the death of mice. For the orthotopic model of spontaneous metastasis, mice were inoculated with 1×10^6^ MDA-MB-231 cells in the No. 4 mammary glands, and tumors were surgically resected when they reached a volume of 300 mm^3^. After resection, bioluminescent imaging was used to monitor metastatic outgrowth.

**Tumor sphere formation assay**

Breast cancer cell lines (MDA-MB-231, MDA-MB-453 and their derivatives) were seeded in 24-well ultralow attachment plates (3473, Corning, New York City, NY, USA) and cultured for 14 days in MEBM (CC-3151, Lonza, Basel, Switzerland) supplemented with 1:50 B27 (17504-044, Thermo Fisher Scientific), 20 ng/ml EGF (PHG0311, Thermo Fisher Scientific), 20 ng/ml bFGF (PHG0261, Thermo Fisher Scientific), 4 μg/ml heparin (H3149-500KU-9, Sigma), 0.5 μg/ml hydrocortisone (H0888, Sigma), 5 μg/ml insulin (I9278, Sigma), and 100 U/ml penicillin/0.1 mg/ml streptomycin.^3^ The cells were cocultured with or without 30 μg/ml collagen I (354236, Corning) or the indicated concentration of antibody. Lung cancer cell lines (H460, H2030 and H1975) were seeded in 24-well ultralow attachment plates and cultured for 7 days in D-MEM/F-12 (12500-062, Thermo Fisher Scientific) supplemented with 1:50 B27, 20 ng/ml EGF, 1×NEAA (11140050, Thermo Fisher Scientific), 10 ng/ml bFGF, 2 μg/ml heparin, and 100 U/ml penicillin/0.1 mg/ml streptomycin.^2^ The cells were cocultured with or without 10 μg/ml collagen I or the indicated concentration of antibody. Melanoma cell lines (A2058 and A375) were seeded in 24-well ultralow attachment plates and cultured for 7 days in D-MEM/F-12 supplemented with 1:50 B27, 20 ng/ml EGF, 10 ng/ml bFGF, and 100 U/ml penicillin /0.1 mg/ml treptomycin.^4^ The cells were cocultured with or without 10 μg/ml collagen I or the indicated concentration of antibody. For serial passage, tumor spheres were collected using 70 μm cell strainers and dissociated with trypsin for 10 min to obtain single-cell suspensions. All tumor spheres in each well were counted with microscopy (Nikon, Tokyo, Japan).

**Analysis of protein and mRNA expression**

For mRNA expression analysis, total RNA from 1×10^5^ cancer cells and tissues were extracted with an RNAprep Pure Cell/Bacteria Kit (DP430, TIANGEN, Beijing, China), and 1 μg of total RNA was reverse transcribed with a ReverTra Ace qPCR RT Kit (FSQ-101, TOYOBO, Osaka, Japan). Q-PCR was performed with TaqMan Gene Expression Assay (Applied Biosystems, Foster City, CA, USA). All quantifications were normalized to endogenous *GAPDH*.

Taqman Gene Expression Assay. NANOG, Hs04260366_g1; POU5F1, Hs04260367_gH; SOX2, Hs01053049_s1; SOX9, Hs01001343_g1; TAZ, Hs00794094_m1; TM4SF1, Hs00371997_m1; GAPDH, Hs99999905_m1.

For flow cytometric analysis, 3×10^5^ cells were resuspended in ice cold FACS buffer (PBS, 10% FBS, 1% sodium azide). Subsequently, cell suspensions were incubated with the indicated antibodies for 30 minutes at 4°C and then analyzed by flow cytometry. The flow cytometry data were analyzed with FlowJo software. The following antibodies were used in FACS: anti-hTM4SF1/L6 (MAB8164, clone: 877621, lot: CIGD0115101, dilution: 1:200, R&D Systems, Canandaigua, NY, USA), PE mouse anti-human CD44 (555479, clone: G44-26, lot: 5191551, dilution: 1:20, BD Pharmingen, San Jose, CA, USA), Alexa Fluor^TM^ 647 mouse anti-human CD24 (561644, clone: ML5, lot: 5288699, dilution: 1:10, BD Pharmingen), PE mouse anti-human CD133 Antibody (130-113-748, clone: 293C3, lot: 5180124056, dilution: 1:50, Miltenyi, Bergisch Gladbach, Germany), Alexa Fluor^TM^ 647 goat anti-mouse IgG (H+L) (A21236, lot: 1887151, dilution: 1:500, Thermo Fisher Scientific).

For flow cytometric analysis of the CD44^high^/CD24^low^ expression level in TM4SF1^low^ and TM4SF1^high^ MDA-MB-231 cells, these cells were labeled with the anti-TM4SF1 antibody followed by the APC-conjugated secondary antibody. After FACS, the cells were passaged for three generations (approximately one week of culture). Before CD44/CD24 expression was detected by FACS analysis, we confirmed that no APC signal remained in either TM4SF1^high^ or TM4SF1^low^ MDA-MB-231 cells, and that the cells maintained TM4SF1 expression.

For immunoblotting, cells were lysed in RIPA buffer (50 mM Tris-HCl pH 7.4, 150 mM NaCl, 1 mM EDTA, 1% Triton X-100, 1% sodium deoxycholate, and 0.1% SDS) supplemented with 1 mM Na_3_VO_4_, phosphatase inhibitor cocktail (B15001, Bimake, Houston, TX, USA), and protease inhibitors (539134, Merck, Darmstadt, Germany). Protein concentrations were measured with the Enhanced BCA Protein Assay Kit (P0010, Beyotime). The proteins were separated by NuPAGE™ 10% Bis-Tris Gel (NP0316BOX, Thermo Fisher Scientific), transferred to a nitrocellulose filter membrane, blocked and tested with the indicated primary antibody and horseradish peroxidase (HRP)-conjugated secondary antibody. The membrane was exposed to an Immobilon® Western Chemiluminescent HRP Substrate (WBKLS0500, Merck). The relative protein expression was quantified by ImageJ. The following antibodies were used in western blots: anti-Flag tag (F3165, clone: M2, lot: SLCC4005, dilution: 1:1000, Sigma), anti-Myc tag (2276, clone: 9B11, lot: 24, dilution: 1:1000, Cell Signaling Technology, Danvers, MA, USA), anti-STAT3 (phospho Y705) (ab76315, clone: Ep2147Y, lot: GR195041-19, dilution: 1:1000, Abcam, Cambridge, UK), anti-STAT3 (9139, clone: 124H6, lot: 7, dilution: 1:1000, Cell Signaling Technology), anti-β-actin (A5316, clone AC-74, lot: 052M4793, dilution 1:5000, Sigma), goat anti-mouse IgG-HRP (SA00001-I, lot: 20000002, dilution 1:20,000, Proteintech, Chicago, IL, USA), and goat anti-rabbit IgG-HRP (sc-2004, lot: F2215, dilution 1: 10,000, Santa Cruz Biotechnology, Dallas, TX, USA).

**Coimmunoprecipitation assay**

For coimmunoprecipitation analysis, cells were lysed in IP-RIPA buffer (50 mM Tris-HCl pH 7.4, 150 mM NaCl, 1 mM EDTA, 1% Triton X-100, 10% glycerol, 0.5 mg/ml BSA, and 0.1% SDS) supplemented with 1 mM Na_3_VO_4_, phosphatase inhibitor cocktail, and protease inhibitors. To immunoprecipitate Flag-tagged protein, total lysates containing 3 mg proteins were incubated with 10 μl Flag M2 affinity gel (A2220, lot: SLCC6965, Sigma) for 12 h at 4°C. After washing three times, the protein–antibody mixture was eluted with Flag peptide (A6001, lot: 11, Apexbio, Houston, TX, USA) for 30 min according to the manufacturer’s protocols. The immune complexes and total lysates were boiled and subjected to western blotting.

**Generation of FC17-7 Antibody**

Female 6- to 8-week-old BALB/c mice were immunized with peptide of TM4SF1 loop1. Following an 8-week boosting protocol, splenocytes were isolated from immunized mice and electrofused with the myeloma cell line SP2/0 to generate hybridoma cells. The hybridomas were subcloned and assayed by ELISA for their ability to secrete immunoglobulin. The monoclonal antibodies were affinity purified on protein G-Sepharose (17-0618-01, GE Healthcare, Chicago, IL, USA) and further characterized. The mouse hybridoma cell strain FC17-7 has been deposited in the China Center for Type Culture Collection. The accession number is C2017110. For antibody binding characterization, FACS binding assays were performed to evaluate the binding of FC17-7 to A549 cells that express high endogenous levels of TM4SF1. FACS analysis were performed by incubating 3×10^5^ cells with FC17-7 at 4°C for 30 min, washed twice in 3% BSA/PBS. The cells were then treated with Alexa Fluor^TM^ 647 goat anti-mouse IgG (H+L) at 4°C for 30 min, washed twice in 3% BSA/PBS and finally analyzed by flow cytometry.

**Histological Analysis**

For the histological analysis, lung samples were fixed in 4% paraformaldehyde solution for 36 h at 4°C, followed by paraffin embedding and sectioning at 6 μm. The sections were stained with hematoxylin (C0107, Beyotime) and eosin (G1001, Servicebio, Wuhan, China) (H&E) according to the standard protocol. Lung metastases were evaluated by microscopic analysis of lung sections.

**Patient samples and immunohistochemical staining**

The BCN963a multiple organ tumor and adjacent normal tissue microarray (TMA) was purchased from Biomax (Derwood, MD, USA). The TMA contained 16 types of organ cancer with matched or unmatched adjacent normal tissue (colon, kidney, liver, prostate, pancreas, esophagus, stomach, rectum, lung, breast, cervix, ovary, bladder, lymph node, skin, and cerebrum). Immunohistochemical staining for human TM4SF1 was performed on paraffin-embedded TMAs with a VECTSTAIN ABC HRP Kit (PK6100, Vectorlabs, Burlingame, CA, USA) and DAB (D5905, Sigma). Antigen retrieval for TM4SF1 staining was performed with citrate buffer pH 6.0. Subsequently, immunohistochemical staining was performed with an anti-TM4SF1 antibody (HPA002823, lot: A75337, Sigma) followed by biotinylated goat anti-rabbit IgG (BA-1000, lot: ZG0818, Vectorlabs). Stained immunohistochemistry specimens were scored with the H-score. Intensity was considered "0" for absent expression, "1+" for weak staining, "2+" for moderate staining, and "3+"for strong staining. The H-score was calculated as follows: H-Score = 0×percentage of absent expression + 1×percentage of weakly stained cells + 2×percentage of moderately stained cells + 3×percentage of strongly stained cells.

**Statistical analysis**

Group sizes for in *vivo* and *in vitro* experiments were selected based on the experiences of intragroup variation. All data analysis and figure plotting were performed using GraphPad Prism (version 8.3.0). Values represent the mean ± s.e.m. values. The numbers of replicates and the approaches to calculate the significant differences are listed in the figure legends. A *P* value less than 0.05 was considered statistically significant for all analyses.

**References**

1. Ponomarev, V. et al. A novel triple-modality reporter gene for whole-body fluorescent, bioluminescent, and nuclear noninvasive imaging. *European Journal of Nuclear Medicine and Molecular Imaging* **31,** 740–751 (2004).

2. She, X., Gao, Y., Zhao, Y., Yin, Y. & Dong, Z. A high-throughput screen identifies inhibitors of lung cancer stem cells. *Biomedicine & Pharmacotherapy* **140,** 111748 (2021).

3. Gao, H. et al. Multi-organ Site Metastatic Reactivation Mediated by Non-canonical Discoidin Domain Receptor 1 Signaling. *Cell* **166,** 47–62 (2016).

4. Chang, X. et al. miR-203 inhibits melanoma invasive and proliferative abilities by targeting the polycomb group gene BMI1. *Biochemical and Biophysical Research Communications* **456,** 361–366 (2015).


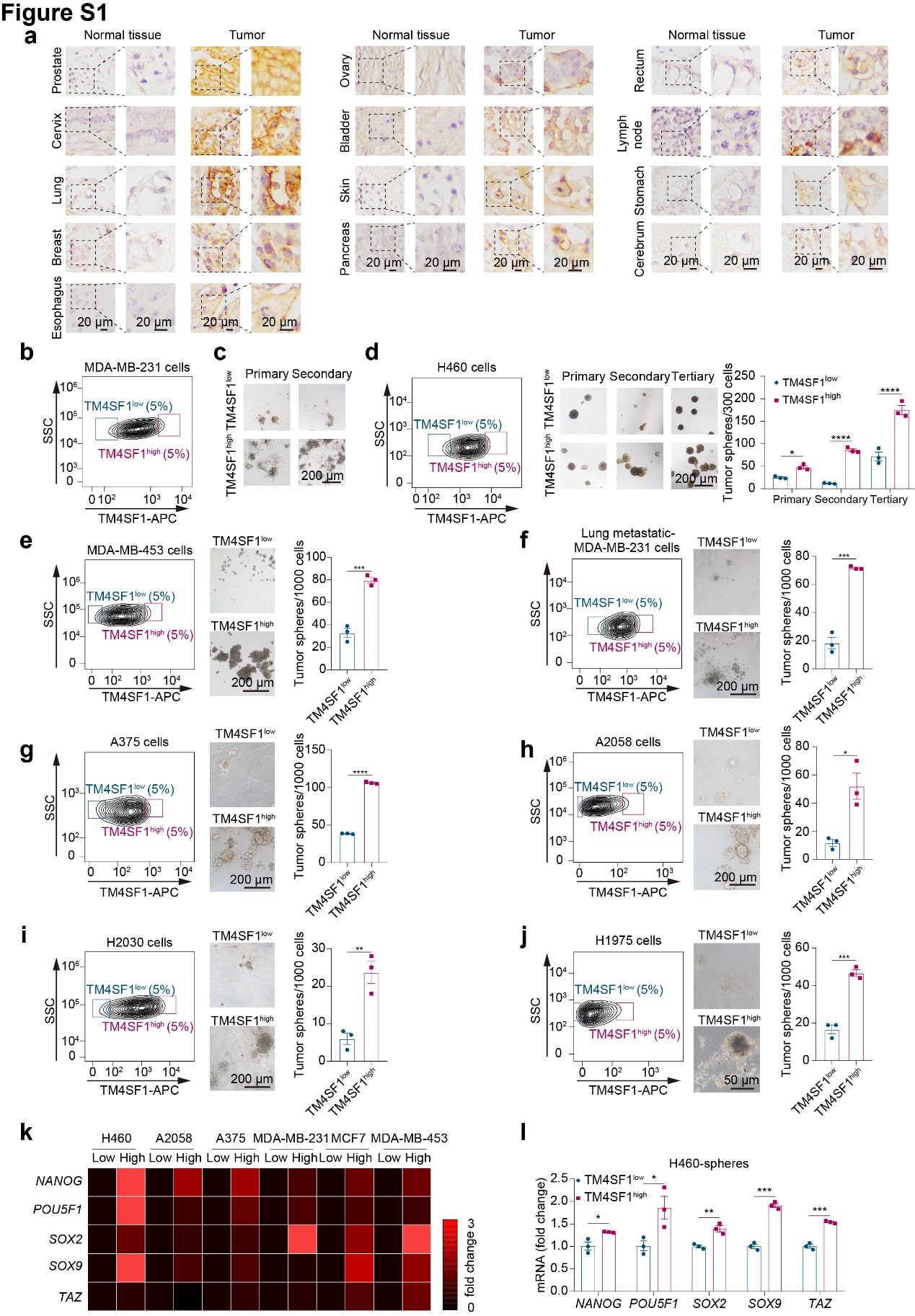


**Figure S1 TM4SF1 is a cell membrane marker of CSCs.**

**a** Representative immunohistochemical images of TM4SF1 expression in clinical samples of 13 types of organ cancer with the adjacent normal tissue (prostate, pancreas, esophagus, stomach, rectum, lung, breast, cervix, ovary, bladder, lymph node, skin, and cerebrum). **b** FACS of live cells was used to separate the TM4SF1^low^ (5%) and TM4SF1^high^ (5%) subpopulations of MDA-MB-231 human breast cancer cells (3 independent experiments). **c** Representative images of spheres. TM4SF1^low^ and TM4SF1^high^ MDA-MB-231 cells (1000 cells/well) were subjected to a tumor sphere assay in medium supplemented with 30 μg/ml collagen I for 14 days. The primary spheres were dissociated and subjected to secondary tumor sphere assays (3 independent experiments). **d** The TM4SF1^low^ (5%) and TM4SF1^high^ (5%) H460 cells (300 cells/well) were subjected to a tumor sphere assay in medium supplemented with 10 μg/ml collagen I for 7 days. The primary spheres were dissociated and subjected to secondary tumor sphere assays. Finally, the secondary tumor spheres were subjected to the same protocol to derive tertiary tumor spheres. Flow cytometric analysis (left panel). Representative images of spheres (middle panel). Statistical analysis (right panel) (3 independent experiments). **e-j** The TM4SF1^low^ (5%) and TM4SF1^high^ (5%) subpopulations of cancer cells (1000 cells/well) were subjected to a tumor sphere assay in medium supplemented with collagen I. Flow cytometric analysis (left panel). Representative images of spheres (middle panel). Statistical analysis (right panel) (3 independent experiments). MDA-MB-453 cells (10 μg/ml collagen I for 14 days) (**e**). Lung metastatic MDA-MB-231 cells (30 μg/ml collagen I for 14 days) (**f**). A375 cells (10 μg/ml collagen I for 7 days) (**g**). A2058 cells (10 μg/ml collagen I for 7 days) (**h**). H2030 cells (10 μg/ml collagen I for 7 days) (**i**). H1975 cells (10 μg/ml collagen I for 7 days) (**j**). **k** qPCR analysis of the mRNA expression levels of five pluripotency factors in TM4SF1^low^ and TM4SF1^high^ H460, A2058, A375, MDA-MB-231 isolated from MDA-MB-231 primary tumors, MDA-MB-453, and MCF7 cells (3 independent experiments). **l** qPCR analysis of the mRNA expression levels of five pluripotency factors in H460 primary spheres from **Fig. S1d** (3 independent experiments). The data are presented as the mean ± s.e.m. values. *P* values were determined by two-way ANOVA with uncorrected Fisher’s LSD test (**d** and **l**) or an unpaired two-tailed Student’s t test with Welch’s correction (**e-j**). **P*<0.05, ***P*<0.01, ****P*<0.001, and *****P*<0.0001.


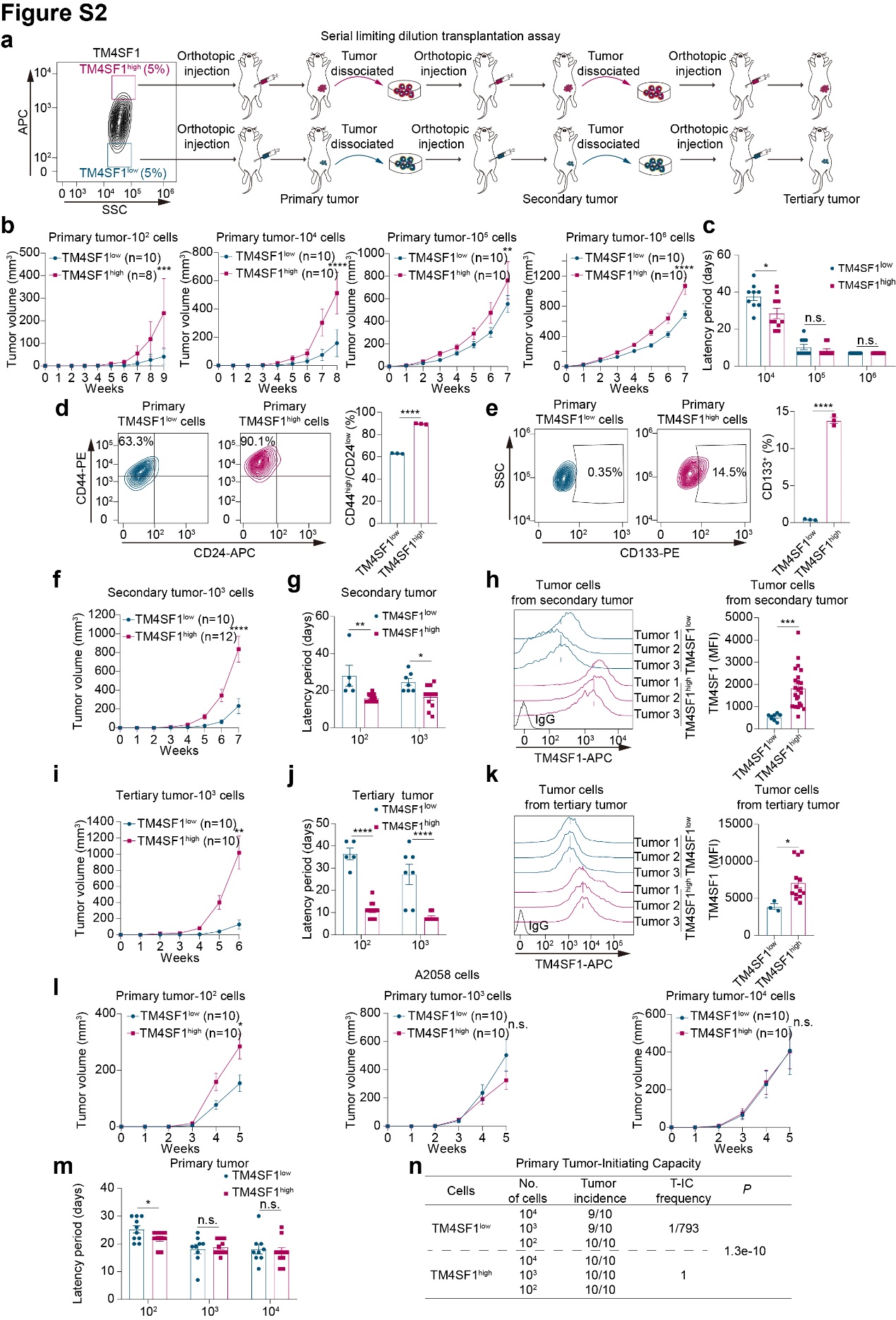


**Figure S2 TM4SF1^high^ cells have the characteristics of CSCs that can be stably maintained and passaged to subsequent generations.**

**a** Schematic of serial limiting dilution transplantation assays. **b**, **c** TM4SF1^low^ and TM4SF1^high^ MDA-MB-231 cells were inoculated into the mammary glands of BALB/c nude mice at the indicated numbers (**b**). Latency period (**c**). **d** Flow cytometric analysis of the CD44^high^/CD24^low^ expression level in TM4SF1^low^ and TM4SF1^high^ primary MDA-MB-231 cells (3 independent experiments). **e** Flow cytometric analysis of the CD133 expression level in TM4SF1^low^ and TM4SF1^high^ primary MDA-MB-231 cells (3 independent experiments). **f-h** Secondary tumor transplantation assay. Tumor volume at the indicated cell number (**f**). Latency period (**g**). Flow cytometric analysis of TM4SF1 expression on the cell membrane of secondary tumors, at least 3 independent experiments (**h**). **i-k** Tertiary tumor transplantation assay. Tumor volume at the indicated cell number (**i**). Latency period (**j**). Flow cytometric analysis of TM4SF1 expression on the cell membrane of tertiary tumors, at least 3 independent experiments (**k**). **l-n** BALB/c nude mice were subcutaneously injected with TM4SF1^low^ and TM4SF1^high^ A2058 cells at the indicated numbers. Tumor volume (**l**). Latency period (**m**). Tumor-initiating capacity (**n**). The n-values denote the number of tumors per group. The data are presented as the mean ± s.e.m. values. *P* values were determined by two-way ANOVA with uncorrected Fisher’s LSD test (**b**, **c**, **f**, **g**, **i**, **j**, **l** and **m**) or an unpaired two-tailed Student’s t-test with Welch’s correction (**d**, **e**, **h** and **k**). **P*<0.05, ***P*<0.01, ****P*<0.001, *****P*<0.0001, and n.s., not significant.


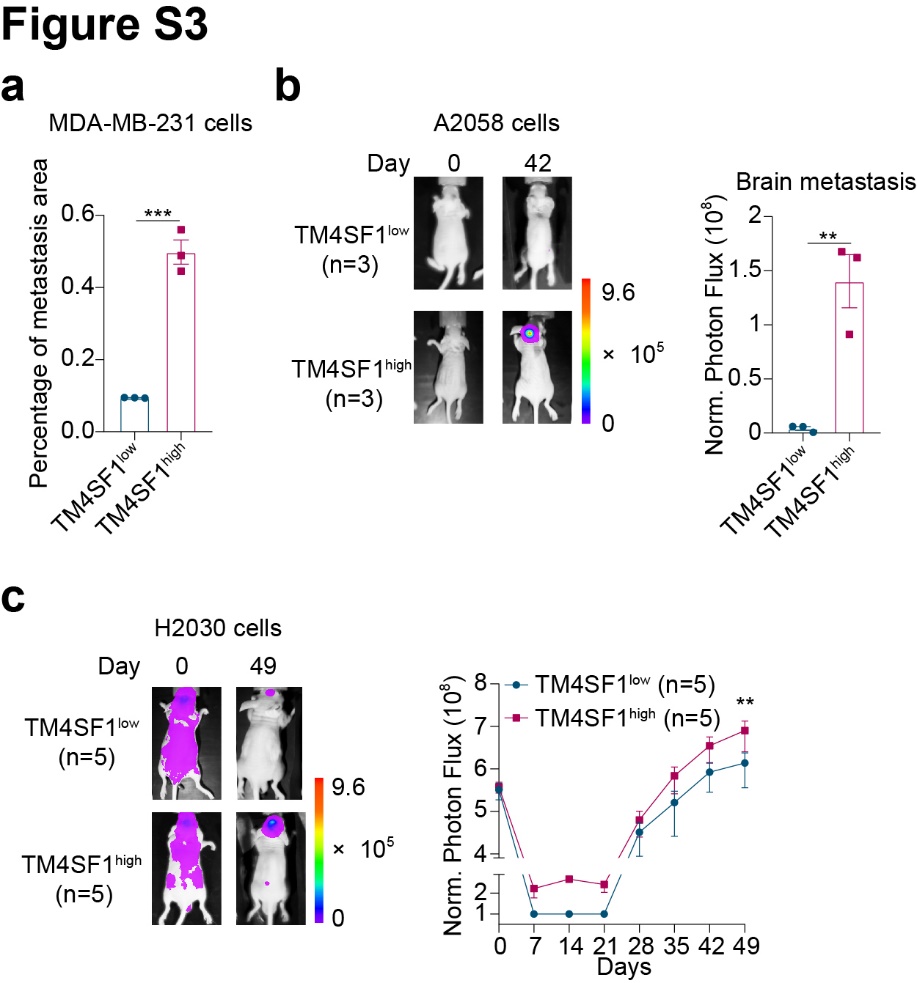


**Figure S3 TM4SF1^high^ cells have higher metastatic ability than TM4SF1^low^ cells.**

**a** Percentage of metastasis area. Lung sections from the MDA-MB-231 primary tumor transplantation assay on Day 56 were subjected to H&E staining (3 independent experiments). **b**, **c** Bioluminescence imaging (left panels) and quantification (right panels) of brain metastases of BALB/c nude mice xenografted with TM4SF1^low^ or TM4SF1^high^ A2058 cells by intracardiac injection (1000 cells) (**b**). Bioluminescence imaging (left panels) and quantification (right panels) of metastases of BALB/c nude mice xenografted with TM4SF1^low^ or TM4SF1^high^ H2030 cells by intracardiac injection (1×10^5^ cells) (**c**). The n-values denote the number of mice per group. The data are presented as the mean ± s.e.m. values. *P* values were determined by an unpaired two-tailed Student’s t test with Welch’s correction (**a** and **b**) or two-way ANOVA with uncorrected Fisher’s LSD test (**c**). ***P*<0.01, and ****P*<0.001.
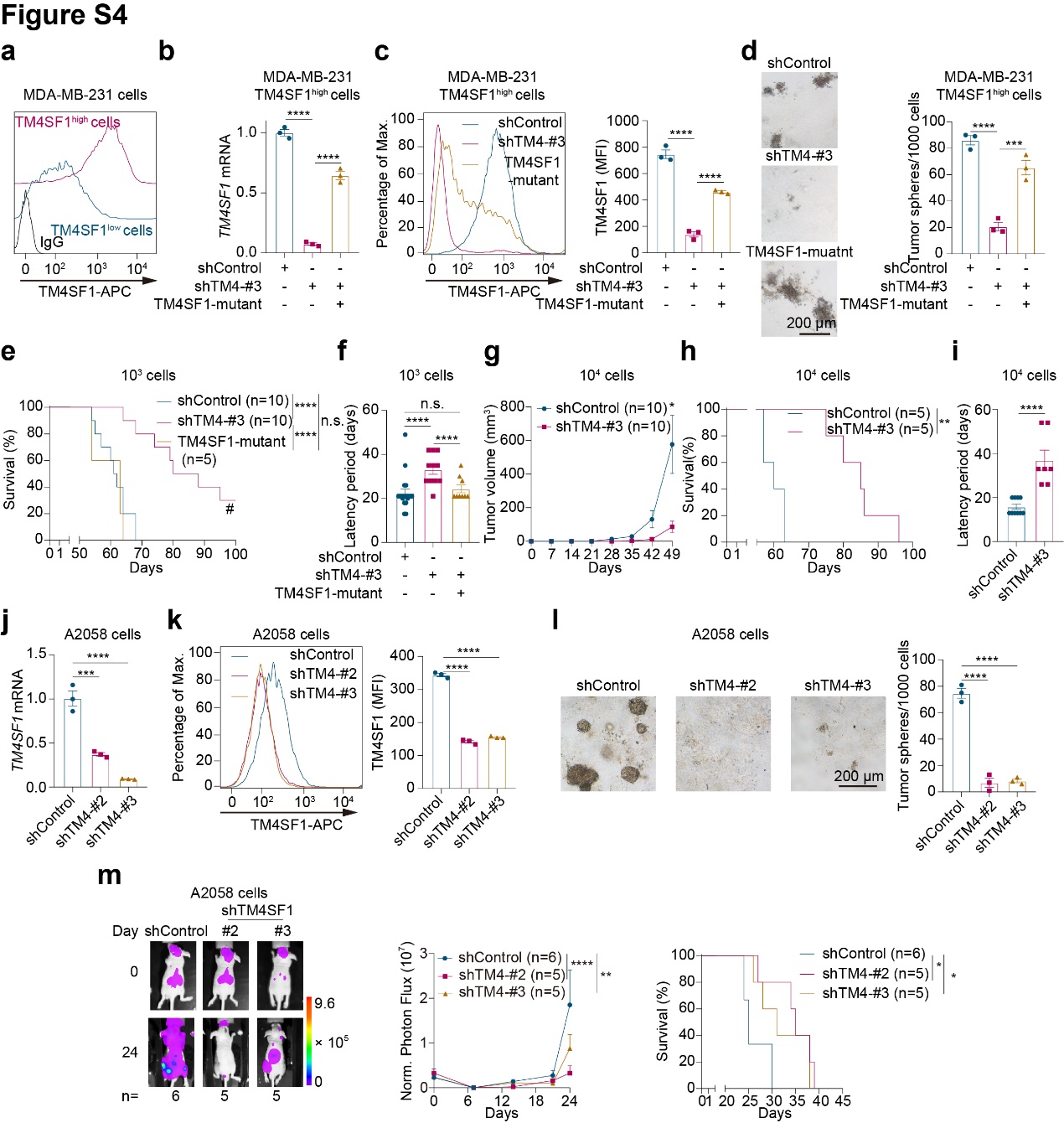


**Figure S4 TM4SF1 is necessary for TM4SF1^high^ cells to possess and maintain the function of CSCs.**

**a** Flow cytometric analysis of TM4SF1 expression on the cell membrane of TM4SF1^low^ and TM4SF1^high^ MDA-MB-231 cells (3 independent experiments). **b**, **c** qPCR analysis (**b**) and flow cytometric analysis (**c**) of the expression of TM4SF1 in shControl, TM4SF1-silenced (shTM4-#3), and shRNA-resistant mutant TM4SF1 cDNA-overexpressing (TM4SF1-mutant) TM4SF1^high^ MDA-MB-231 cells (3 independent experiments). **d** Tumor sphere formation assay for shControl, shTM4-#3, and TM4SF1-mutant cells (1000 cells/well) with collagen I (30 μg/ml) for 14 days (3 independent experiments). **e-i** shControl, shTM4-#3 and TM4SF1-mutant cells were inoculated into the mammary glands of BALB/c nude mice at the indicated numbers. Overall survival (The n-values denote the number of mice per group.) (**e**, **h**). Tumor volume (The n-values denote the number of tumors per group.) (**g**). Latency period (**f**, **i**). **j**, **k** qPCR analysis (**j**) and flow cytometric analysis (**k**) of the expression of TM4SF1 in shControl and TM4SF1-silenced A2058 cells (shTM4-#2, shTM4-#3) (3 independent experiments). **l** Tumor sphere formation assay for shControl and TM4SF1-silenced A2058 cells (1000 cells/well) with 10 μg/ml collagen I for 7 days (3 independent experiments). **m** Bioluminescence imaging (left panels) and quantification (middle panels) of metastases and overall survival (right panels) of BALB/c nude mice xenografted with shControl or TM4SF1-silenced A2058 cells by intracardiac injection (3×10^4^ cells) (The n-values denote the number of mice per group). The data are presented as the mean ± s.e.m. values. *P* values were determined by one-way ANOVA with uncorrected Fisher’s LSD test (**b-d**, **f** and **j-l**), the log-rank test (**e**, **h** and **m**) or two-way ANOVA with uncorrected Fisher’s LSD test (**g** and **m**) or an unpaired two-tailed Student’s t test with Welch’s correction (**i**). **P*<0.05, ***P*<0.01, ****P*<0.001, *****P*<0.0001, n.s., not significant, and #, end of the experiment.


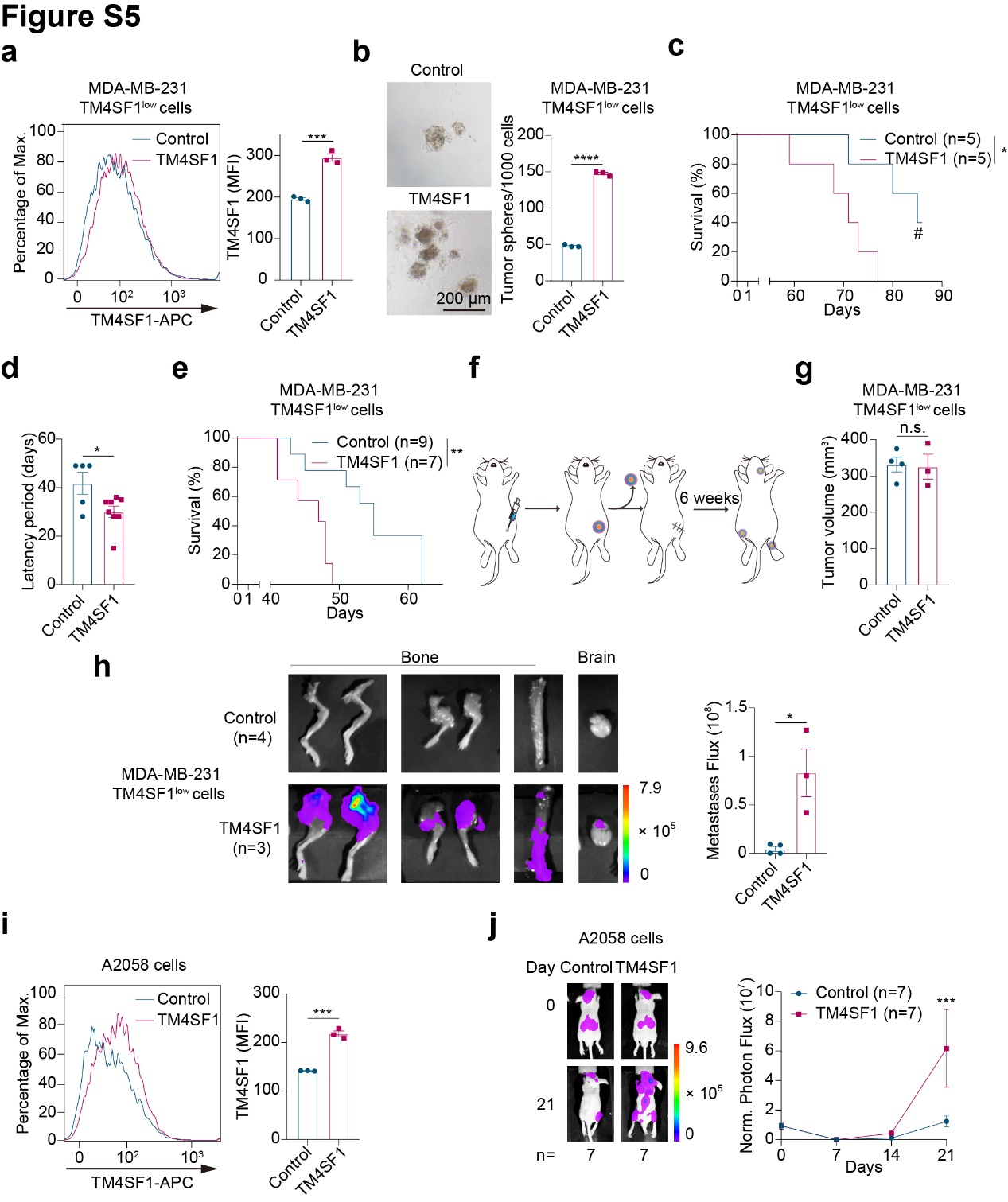


**Figure S5 High expression of TM4SF1 is a sufficient condition for the possession and maintenance of CSC characteristics by cancer cells.**

**a** Flow cytometric analysis of the expression of TM4SF1 in control and TM4SF1-overexpressing TM4SF1^low^ MDA-MB-231 cells (3 independent experiments). **b** Tumor sphere formation assay for control and TM4SF1-overexpressing TM4SF1^low^ MDA-MB-231 cells (1000 cells/well) with collagen I (30 μg/ml) for 14 days (3 independent experiments). **c**, **d** Control and TM4SF1-overexpressing TM4SF1^low^ MDA-MB-231 cells (1000 cells) were inoculated into the mammary glands of BALB/c nude mice at the indicated numbers. Overall survival (**c**). Latency period (**d**). **e** Overall survival of BALB/c nude mice xenografted with control or TM4SF1-overexpressing TM4SF1^low^ MDA-MB231 cells by intracardiac injection (1×10^5^ cells). **f-h** Control and TM4SF1-overexpressing TM4SF1^low^ MDA-MB-231 cells (1×10^6^ cells) were inoculated into the mammary glands of BALB/c nude mice, and then tumors were removed after reaching a volume of 300 mm^3^. TM4SF1-overexpressing TM4SF1^low^ MDA-MB-231 primary tumors were surgically resected on Day 21, while TM4SF1^low^ MDA-MB-231 primary tumors were surgically resected on Day 28. Multiorgan metastases were detected by bioluminescent imaging. Schematic of the orthotopic spontaneous metastasis model (**f**). Tumor volume (**g**). Bioluminescence imaging (left panels) and quantification (right panels) of metastases (**h**). **i** Flow cytometric analysis of the expression of TM4SF1 in control and TM4SF1-overexpressing A2058 cells (3 independent experiments). **j** Bioluminescence imaging (left panels) and quantification (right panels) of metastases of BALB/c nude mice xenografted with control or TM4SF1-overexpressing A2058 cells by intracardiac injection (3×10^4^ cells). The n-values denote the number of mice per group. The data are presented as the mean ± s.e.m. values. *P* values were determined by an unpaired two-tailed Student’s t test with Welch’s correction (**a**, **b**, **d**, **g**, **h** and **i**) or the log-rank test (**c** and **e**) or two-way ANOVA with uncorrected Fisher’s LSD test (**j**). **P*<0.05, ***P*<0.01, ****P*<0.001, *****P*<0.0001, n.s., not significant, and #, end of the experiment.


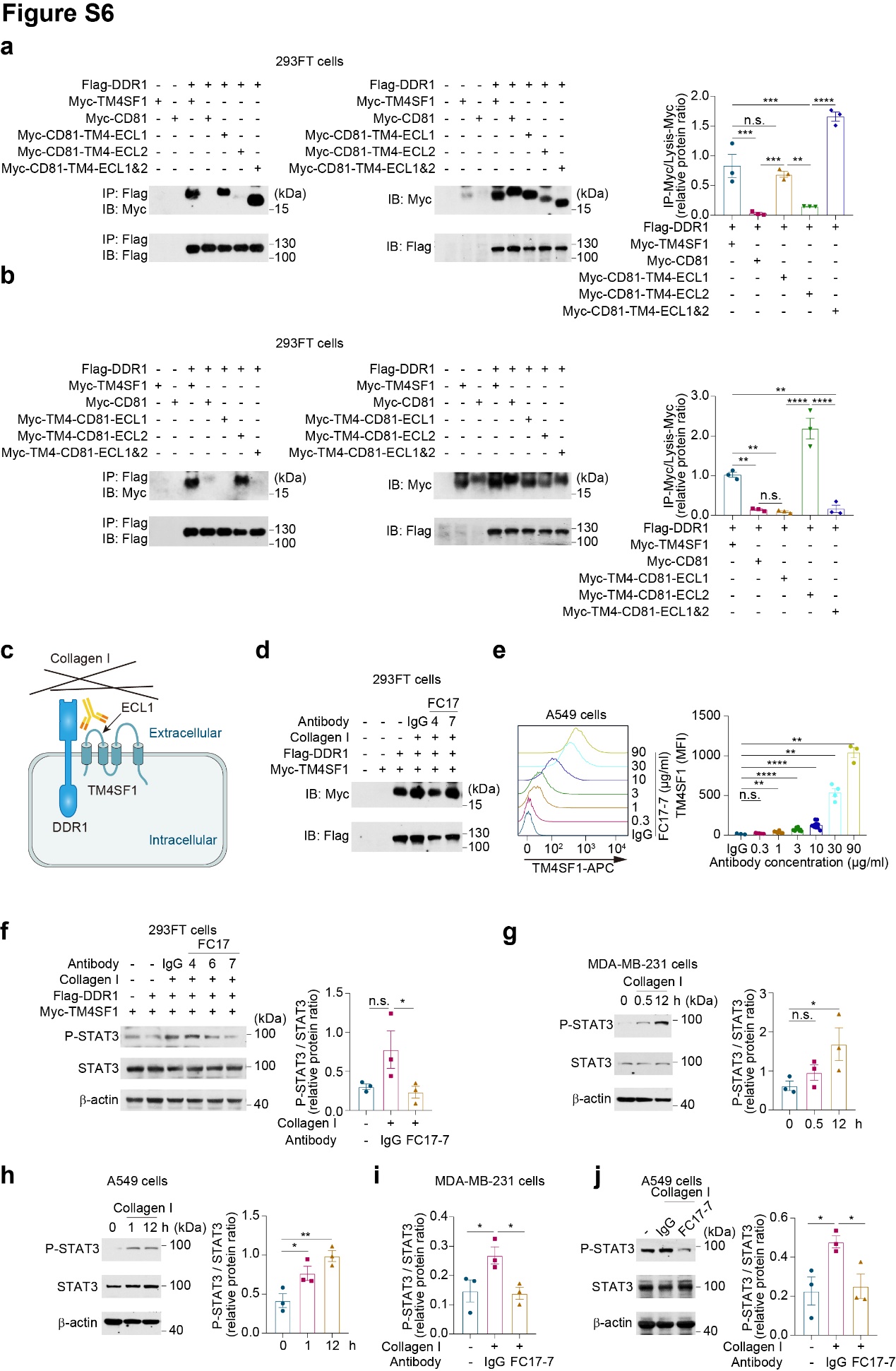


**Figure S6 FC17-7 inhibits the activation of JAK2-STAT3 signaling by blocking the interaction between ECL1 of TM4SF1 and DDR1.**

**a** 293FT cells were cotransfected with Flag-DDR1 together with Myc-TM4SF1, Myc-CD81, Myc-CD81-TM4-ECL1 (extracellular loop 1 of CD81 replaced by extracellular loop 1 of TM4SF1), Myc-CD81-TM4-ECL2 (extracellular loop 2 of CD81 replaced by extracellular loop 2 of TM4SF1), Myc-CD81-TM4-ECL1&2 (both extracellular loops 1 and 2 of CD81 replaced together by extracellular loops 1 and 2 of TM4SF1), or empty vector and were coIP with Flag M2 affinity gel. Flag coIP and total lysates were subjected to IB with the indicated antibodies (3 independent experiments). **b** 293FT cells were cotransfected with Flag-DDR1 together with Myc-TM4SF1, Myc-CD81, Myc-TM4-CD81-ECL1 (extracellular loop 1 of TM4SF1 replaced by extracellular loop 1 of CD81), Myc-TM4-CD81-ECL 2 (extracellular loop 2 of TM4SF1 replaced by extracellular loop 2 of CD81), Myc-TM4-CD81-ECL 1&2 (both extracellular loops 1 and 2 of TM4SF1 replaced together by extracellular loops 1 and 2 of CD81), or empty vector and were coIP with Flag M2 affinity gel. Flag coIP and total lysates were subjected to IB with the indicated antibodies (3 independent experiments). **c** Schematic of the strategy to acquire monoclonal antibodies targeting extracellular loop 1 of TM4SF1. **d** 293FT cells transfected with Myc-TM4SF1, together with Flag-DDR1, or empty vector were treated with 10 μg/ml antibodies and 30 μg/ml collagen I for 6 hr and coIP with Flag M2 affinity gel. Lysates were subjected to IB with the indicated antibodies. **Fig. 1t** shows the IP results, and **Fig. S6d** shows the total lysate results (3 independent experiments). **e** Flow cytometric analysis of the binding capacity of FC17-7 in A549 cells. Mean fluorescence (MFI) values were plotted versus antibody concentration (at least 3 independent experiments). **f** 293FT cells transfected with Myc-TM4SF1, together with Flag-DDR1, or empty vector were treated with 10 μg/ml the indicated antibodies and 30 μg/ml collagen I for 12 hr. Total lysates were subjected to IB with the indicated antibodies (3 independent experiments). **g**, **h** MDA-MB-231 cells and A549 cells were treated with collagen I for the indicated times. Total lysates were subjected to IB with the indicated antibodies [MDA-MB-231 (10 μg/ml collagen I) (**g**), A549 (30 μg/ml collagen I) (**h**)] (3 independent experiments). **i** Quantification of relative protein expression. MDA-MB-231 cells were treated with 10 μg/ml collagen I, alone or in combination with 10 μg/ml FC17-7 for 12 hr (3 independent experiments). Additionally, see **Fig. 1u** for images of IB. **j** A549 cells were treated with 30 μg/ml collagen I, alone or in combination with 10 μg/ml FC17-7 for 12 hr. Total lysates were subjected to IB with the indicated antibodies (3 independent experiments). The data are presented as the mean ± s.e.m. values. *P* values were determined by one-way ANOVA with uncorrected Fisher’s LSD test (**a, b,** and **e**-**j**). **P*<0.05, ***P*<0.01, ****P*<0.001, *****P*<0.0001, and n.s., not significant.


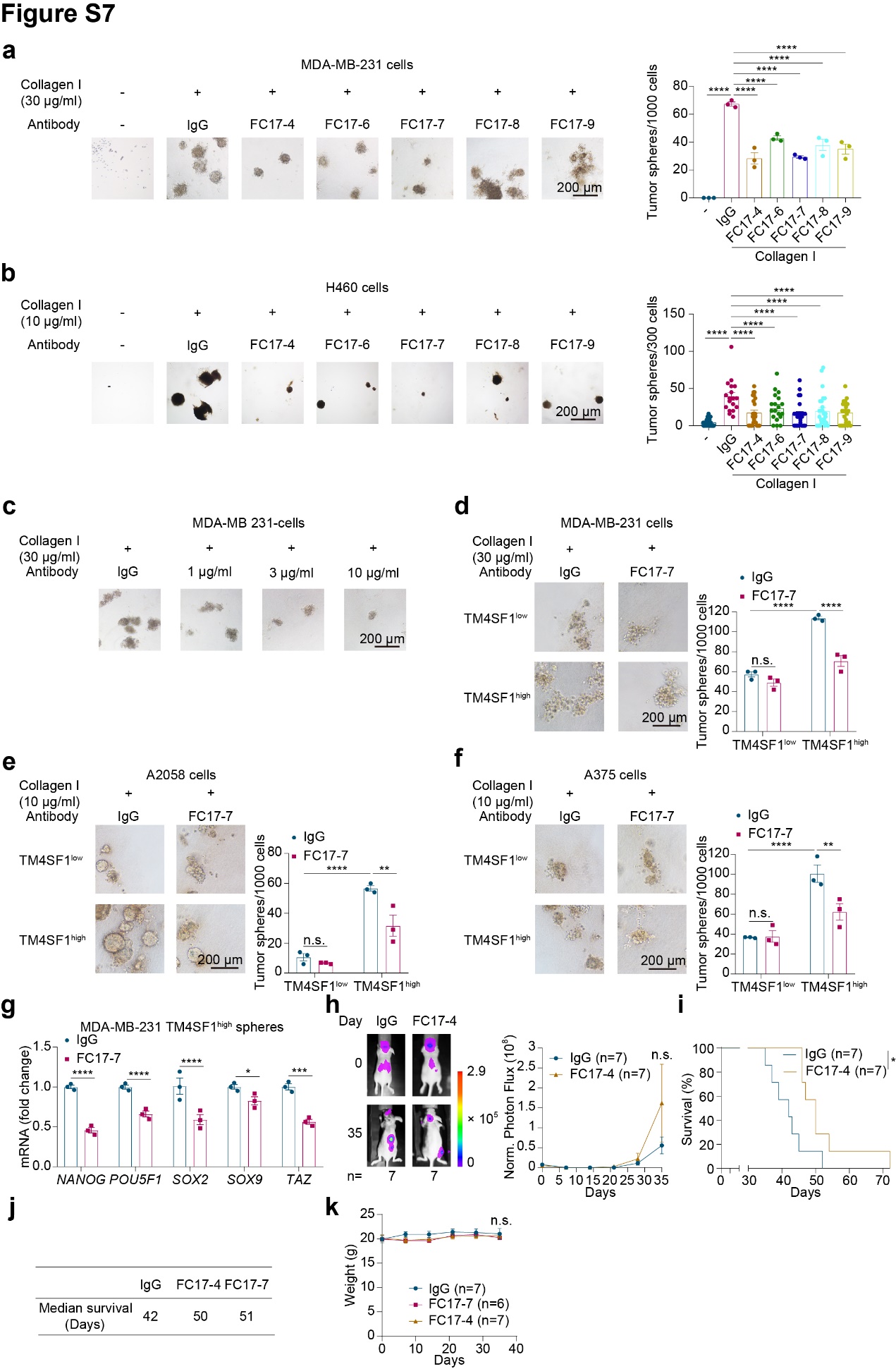


**Figure S7** **FC17-7 inhibits the function of CSCs *in vitro* and *in vivo*.**

**a** Tumor sphere formation assay for MDA-MB-231 cells (1000 cells/well) cultured with IgG or monoclonal antibodies targeting TM4SF1 (10 μg/ml) and stimulated with collagen I (30 μg/ml) for 14 days (3 independent experiments). **b** Tumor sphere formation assay for H460 cells (300 cells/well) cultured with IgG or monoclonal antibodies targeting TM4SF1 (10 μg/ml) and stimulated with collagen I (10 μg/ml) for 7 days (at least 3 independent experiments). **c** Representative images of spheres for MDA-MB-231 cells cultured with IgG or FC17-7 at the indicated concentration and stimulated with collagen I (30 μg/ml) for 14 days (3 independent experiments). **d** Tumor sphere formation assay for TM4SF1^low^ and TM4SF1^high^ MDA-MB-231 cells (1000 cells/well) cultured with IgG or FC17-7 (10 μg/ml) and stimulated with collagen I (30 μg/ml) for 14 days (3 independent experiments). **e**, **f** Tumor sphere formation assay for TM4SF1^low^ and TM4SF1^high^ melanoma cells (1000 cells/well) cultured with IgG or FC17-7 (10 μg/ml) and stimulated with collagen I (10 μg/ml) for 7 days. A2058 cells (**e**), A375 cells (**f**) (3 independent experiments). **g** qPCR analysis of the mRNA expression levels of five pluripotency factors in TM4SF1^high^ MDA-MB-231 spheres. Cells were treated with 30 μg/ml collagen I, alone or in combination with 10 μg/ml FC17-7 for 14 days (3 independent experiments). **h-k** MDA-MB-231 cells (1×10^5^ cells) were intracardially injected into BALB/c nude mice. Metastases throughout the whole body were detected by bioluminescent imaging. The indicated antibodies were administered intraperitoneally once every three days (FC17-7) or every other day (FC17-4) from day −1 to the day the mice were dead. Representative images of cells treated with FC17-4 (left panel). Relative photon flux treated with FC17-4 (right panel) (**h**). Overall survival of FC17-4 (**i**). Median survival (**j**). The weights of the mice (**k**). The n-values denote the number of mice per group. The data are presented as the mean ± s.e.m. values. *P* values were determined by one-way ANOVA with uncorrected Fisher’s LSD test (**a** and **b**), two-way ANOVA with uncorrected Fisher’s LSD test (**d-h**, **k**) or the log-rank test (**i**). **P*<0.05, ***P*<0.01, ****P*<0.001, *****P*<0.0001, and n.s., not significant.
